# Amitriptyline interferes with autophagy-mediated clearance of protein aggregates via inhibiting autophagosome maturation in neuronal cells

**DOI:** 10.1038/s41419-020-03085-6

**Published:** 2020-10-17

**Authors:** Yoonjung Kwon, Yeojin Bang, Soung-Hee Moon, Aeri Kim, Hyun Jin Choi

**Affiliations:** College of Pharmacy and Institute of Pharmaceutical Sciences, CHA University, Pocheon, Gyeonggi-Do 11160 Republic of Korea

**Keywords:** Cellular neuroscience, Neurodegeneration

## Abstract

Amitriptyline is a tricyclic antidepressant commonly prescribed for major depressive disorders, as well as depressive symptoms associated with various neurological disorders. A possible correlation between the use of tricyclic antidepressants and the occurrence of Parkinson’s disease has been reported, but its underlying mechanism remains unknown. The accumulation of misfolded protein aggregates has been suggested to cause cellular toxicity and has been implicated in the common pathogenesis of neurodegenerative diseases. Here, we examined the effect of amitriptyline on protein clearance and its relevant mechanisms in neuronal cells. Amitriptyline exacerbated the accumulation of abnormal aggregates in both in vitro neuronal cells and in vivo mice brain by interfering with the (1) formation of aggresome-like aggregates and (2) autophagy-mediated clearance of aggregates. Amitriptyline upregulated LC3B-II, but LC3B-II levels did not increase further in the presence of NH_4_Cl, which suggests that amitriptyline inhibited autophagic flux rather than autophagy induction. Amitriptyline interfered with the fusion of autophagosome and lysosome through the activation of PI3K/Akt/mTOR pathway and Beclin 1 acetylation, and regulated lysosome positioning by increasing the interaction between proteins Arl8, SKIP, and kinesin. To the best of our knowledge, we are the first to demonstrate that amitriptyline interferes with autophagic flux by regulating the autophagosome maturation during autophagy in neuronal cells. The present study could provide neurobiological clue for the possible correlation between the amitriptyline use and the risk of developing neurodegenerative diseases.

## Introduction

Amitriptyline is a tricyclic antidepressant used to manage major depressive disorders, anxiety disorders, and other mental illnesses. Antidepressive activity of amitriptyline is associated with the increase in synaptic neurotransmission of serotonin and norepinephrine by blocking their neuronal reuptake from the synapse in the central nervous system^1^. Approximately, 40–80% of patients with neurodegenerative diseases suffer from depressive symptoms^2–5^. Amitriptyline could be used to treat depression and anxiety symptoms associated with neurodegenerative diseases including Parkinson’s disease (PD). Patients who received amitriptyline were reported to ameliorate PD-associated depressive symptoms, and delay the need for dopamine therapy as compared with patients who did not take the drug^6,7^. On the other hand, its chronic use has been demonstrated to be a risk factor for neurodegenerative diseases as evidenced by tricyclic antidepressants-induced increase in neuronal cell death in a dorsal root ganglion cell culture model^8^, PD-related neurotoxicity and movement disorders caused by its long-term use in an in vivo model^9^, and development of drug-induced PD by antidepressants^10^. Despite these evidences, there is critical knowledge gap in understanding the underlying mechanism by which amitriptyline causes neurotoxicity.

Protein quality control is crucial for maintaining cellular homeostasis and the functionality of eukaryotic cells. Reactive oxygen species are increased by environmental factors and intracellular metabolism, which cause the accumulation of abnormally high levels of damaged proteins in cells^11,12^. In addition, intracellular pathways responsible for protein homeostasis gradually decrease with aging^13^. Because neurons generally do not replicate, they are more vulnerable to the accumulation of toxic protein aggregates and damaged organelles than nonneuronal cells^14,15^. Therefore, the dysregulation of protein quality control is closely associated with an increased risk of developing neurodegenerative diseases^16–18^. The abnormal accumulation of misfolded proteins such as β-amyloid/tau, α-synuclein, and huntingtin is a characteristic pathology of Alzheimer’s disease, PD, and Huntington’s disease, respectively^19–22^.

There are two main intracellular protein clearance systems maintaining protein homeostasis: ubiquitin–proteasome system and autophagy. Autophagy is a degradation and recycling system targeting damaged or dysfunctional protein complexes and intracellular organelles by delivering them into lysosome, and is upregulated during stressful conditions including the accumulation of misfolded proteins^23,24^. In mammalian cells, there are three major types of autophagy: chaperone-mediated autophagy, microautophagy, and macroautophagy^25^. During chaperon-mediated autophagy, chaperones identify protein containing KFERQ-like sequence, associate them to the lysosomal membrane protein LAMP-2A, and translocate of the protein cargo into the lysosome. In microautophagy, the direct uptake of cargo protein is occurred through sequestration of the lysosomal membrane. Macroautophagy is the best studied, and has gained attention in the field of adult-onset neurodegeneration^26^. Macroautophagy, hereafter referred to as autophagy, begins with the formation of a double-membrane structure called phagophore from intracellular membrane components. During this process, microtubule-associated protein light chain 3 (LC3)-I is lipidated to LC3-II, then localized to the phagophore membrane. Autophagy adaptor p62/SQSTM1 (p62) recruits polyubiquitinated materials and binds to ubiquitinated cargoes as well as LC3, thereby targeting the autophagosome and facilitating clearance of ubiquitinated proteins. After the fusion of the autophagosome with lysosome, the substances are degraded by lysosomal enzymes^27–29^. The balance between autophagosome formation and lysosomal degradation is critical for autophagy-mediated protein clearance. Therefore, the dysregulation of autophagy induction and/or autophagic flux results in the abnormal accumulation of protein aggregates and cytotoxicity.

The purpose of this study is to evaluate whether amitriptyline causes abnormal accumulation of protein aggregates in neuronal cells and whether it dysregulates autophagy function. We also determined the specific step in the autophagy process regulated by amitriptyline, as well as its signaling mechanism. Because autophagy is primarily responsible in preventing abnormal protein accumulation in neuronal cells, this study demonstrating the association between the use of amitriptyline and the interference of autophagy-mediated protein clearance might provide new insight into the risk of neurodegenerative diseases.

## Results

### Amitriptyline increases protein aggregates in neuronal cells and mice brain

**Fig. 1:**
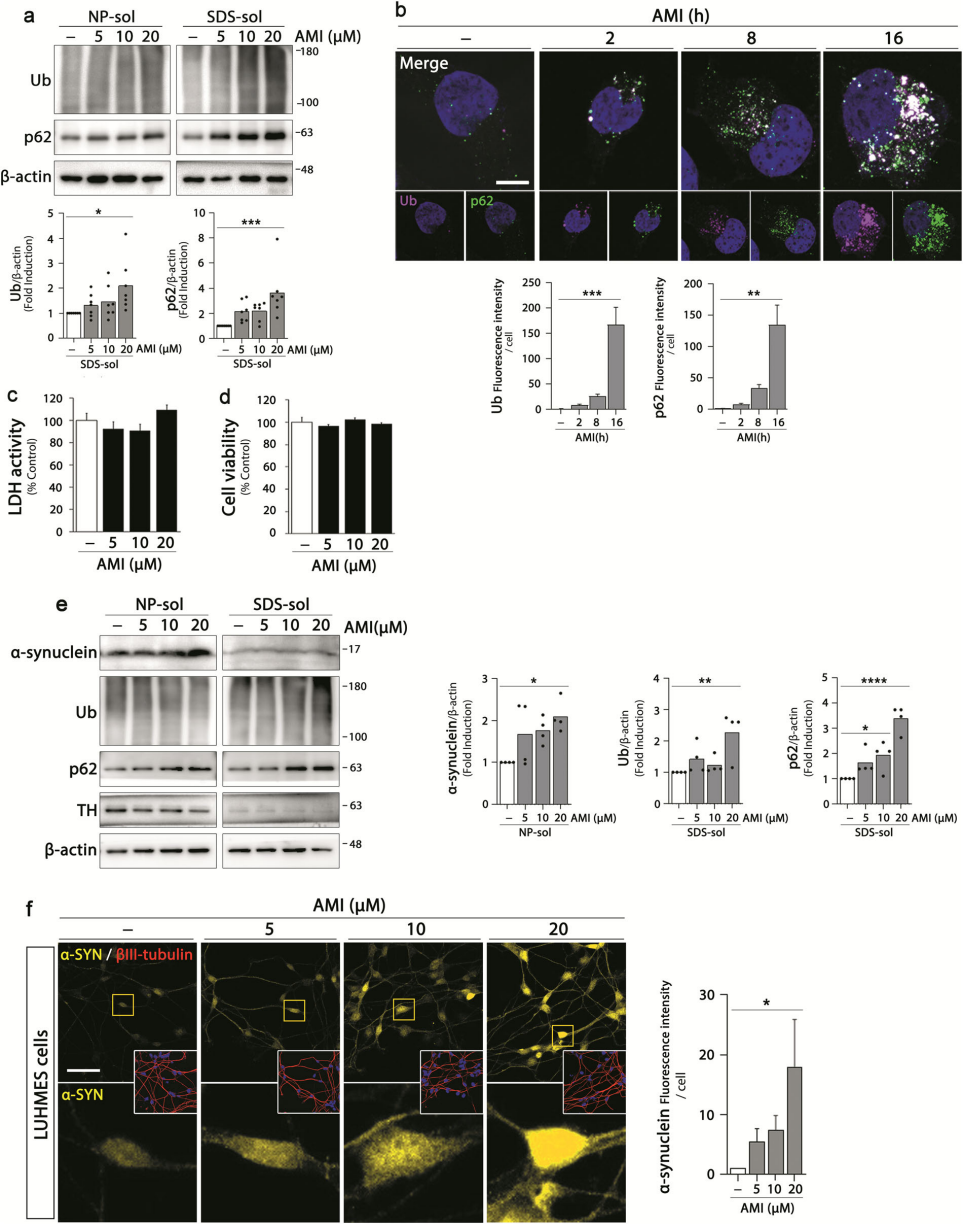
Effect of amitriptyline on protein accumulation in neuronal cells. **a** SH-SY5Y cells were differentiated with 10 μM retinoic acid and treated with amitriptyline at concentrations of 5, 10, or 20 μM for 24 h. Ubiquitin and p62 protein levels in the NP- and SDS-soluble fractions were analyzed by western blotting. Protein levels in the SDS-fraction were quantified by densitometric analysis (*n* = 7). **b** SH-SY5Y cells were treated with amitriptyline at 20 μM for 2, 8, and 16 h and ubiquitin (pink) and p62 (green) levels were analyzed by immunofluorescence. DAPI staining (blue) represented the nuclei. Scale bar corresponds to 10 μm. Quantification of ubiquitin and p62 levels in **b** is shown in the below panel. (*n* = 15–20) **c**, **d** SH-SY5Y cells were treated amitriptyline at 5, 10, and 20 μM for 24 h, and the extent of cell death was assessed by LDH assay (**c**) and MTT assay (**d**) (mean ± S.E.M., *n* = 11–12). **e** LUHMES cells were differentiated as described in “Materials and methods” and treated with amitriptyline at concentrations of 5, 10, or 20 μM for 24 h. Alpha-synuclein, ubiquitin, p62, and tyrosine hydroxylase levels in the NP- and SDS-soluble fractions were analyzed by western blotting. Protein levels in the NP-soluble (α-synuclein) and SDS-soluble (ubiquitin and p62) fractions were quantified by densitometric analysis (*n* = 4). **f** LUHMES cells were treated with amitriptyline at 5, 10, or 20 μM for 24 h, and immunostained with α-synuclein (yellow) and βIII-tubulin (red) antibodies. DAPI staining (blue) represented the nuclei. Scale bar corresponds to 50 μm. Quantification of α-synuclein immunoreactivity in **f** was shown in the right panel. (*n* = 100–150) *P < 0.05, **P < 0.01, ***<0.001, ****P < 0.0001. AMI amitriptyline, NP-sol NP-soluble fraction, SDS-sol SDS-soluble fraction, Ub ubiquitin, α-SYN α-synuclein, TH tyrosine hydroxylase.

The effect of amitriptyline on the accumulation of protein aggregates in neuronal cells was examined by using all-trans retinoic acid-induced differentiated SH-SY5Y cells. We differentiated SH-SY5Y cells for 3 days and then treated with amitriptyline at a concentration range of 5–20 μM for additional 24 h, which concentrations were often used in in vitro studies on amitriptyline effects^9,30,31^. To evaluate the effect of amitriptyline on aggregates accumulation, immunoblot analyses with ubiquitin and p62 were performed on NP- and sodium dodecyl sulfate (SDS)-soluble cell fractions, respectively (Fig. 1a). The accumulation of both ubiquitin polymer (oligomer) and p62 tended to increase with increasing concentration of amitriptyline in SDS-soluble fractions of cells, and a significant increase was shown at 20 μM. We also performed immunostaining of amitriptyline-treated SH-SY5Y cells with ubiquitin and p62 antibodies and analyzed the intracellular levels and distribution pattern under confocal microscope. Ubiquitin and p62 immunoreactivities were significantly increased and largely colocalized with each other after 16 h (Fig. 1b). Amitriptyline-induced increase in ubiquitin and p62 was also detected at 8 h, but not significant. Moreover, there was less colocalization of ubiquitin and p62, which indicated that amitriptyline-induced protein aggregates were ubiquitinated and linked to p62 over time. Amitriptyline was not cytotoxic to differentiated SH-SY5Y cells in the given concentration range (Fig. 1c, d).

To verify the effect of amitriptyline on the protein accumulation in more pathophysiologically relevant in vitro model, we examined the amitriptyline effects in differentiated Lund human mesencephalic (LUHMES) cells. LUHMES cells are human embryonic neuronal precursor cells and differentiated into human dopaminergic neurons, which characterized by increased expression of dopaminergic neuronal markers^32,33^. Because LUHMES cells express α-synuclein and the accumulation of endogenous α-synuclein aggregate is associated with PD pathology^33^, we also evaluated whether accumulation of α-synuclein was induced by amitriptyline. LUHMES cells were differentiated for 5 days and treated with amitriptyline for additional 24 h. The expression of dopaminergic neuronal marker was confirmed by immunoblotting against tyrosine hydroxylase (Fig. 1e). Similar to SH-SY5Y cells, significant increase in ubiquitin and p62 proteins was detected in SDS-soluble fractions of amitriptyline (20 μM)-treated LUHMES cells (significant increase in p62 was shown even at 10 μM). Moreover, amitriptyline caused accumulation of α-synuclein in NP-soluble fractions. Confocal images also showed amitriptyline-induced accumulation of α-synuclein in LUHMES cells (Fig. 1f) and primary ventral mesenchymal (VM) dopaminergic neurons (Supplementary Fig. S1a). Taken together, these data indicate that amitriptyline induces accumulation of abnormal proteins in neuronal cells.

**Fig. 2:**
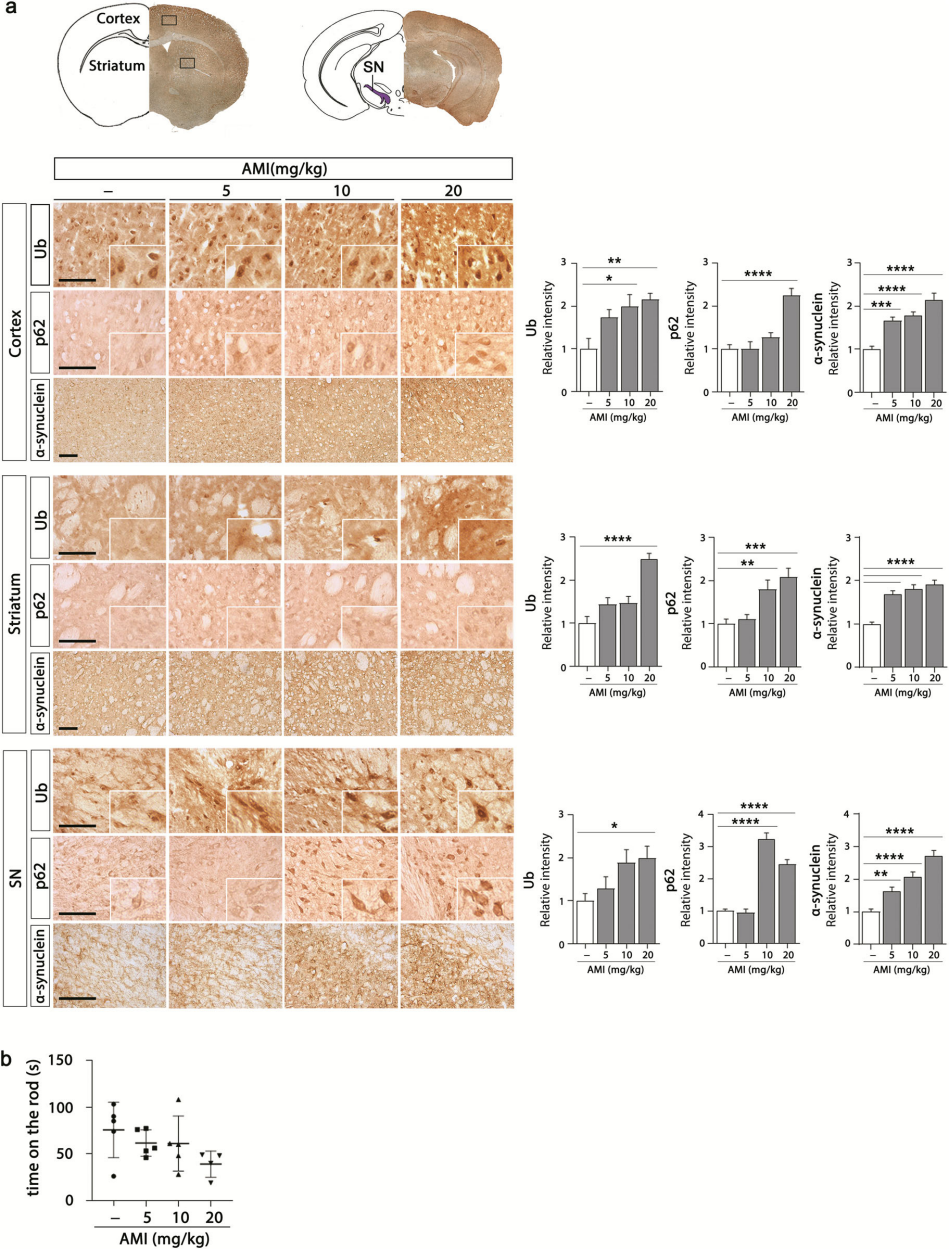
Effect of amitriptyline on protein accumulation in mice brain. **a** Mice were injected with amitriptyline (5, 10, and 20 mg/kg, i.p., *n* = 5) for 30 d, and immunohistochemistry against anti-ubiquitin, anti-p62, and anti-α-synuclein was performed in the cortex, striatum, and SN of mice brain. Immunoreactivities were quantified by densitometric analysis. Scale bar corresponds to 100 μm. **b** Motor dysfunction was assessed by the rotarod test. The latency to fall from a rotating rod was automatically recorded (*n* = 4–5). *P < 0.05, **P < 0.01, ***<0.001, ****P < 0.0001. AMI amitriptyline, SN subsantia nigra, Ub ubiquitin.

Next, we examined the effect of chronic exposure to amitriptyline on the accumulation of protein aggregates in the brain of mice. We injected amitriptyline (i.p. daily for 30 days; 5, 10, and 20 mg/kg) to male C57BL/6 mice, and evaluated ubiquitin, p62, and α-synuclein levels of the cortex, striatum, and substantia nigra (SN) by immunohistochemistry. As shown in Fig. 2a, the significantly increased accumulation of ubiquitin, p62, and α-synuclein was detected in amitriptyline-treated mice brain. Significantly increase in α-synuclein was shown by relatively low doses of amitriptyline (even at 5 mg/kg). To verify whether these conditions caused motor dysfunction, we examined motor coordination of mice by using rotarod test. The exposure of amitriptyline under this condition tended to decrease the motor function of the mice (Fig. 2b). These results clearly show that amitriptyline causes the accumulation of protein aggregates in in vivo models like as in vitro.

### Amitriptyline aggravates a MG132-induced accumulation of aggregates

**Fig. 3:**
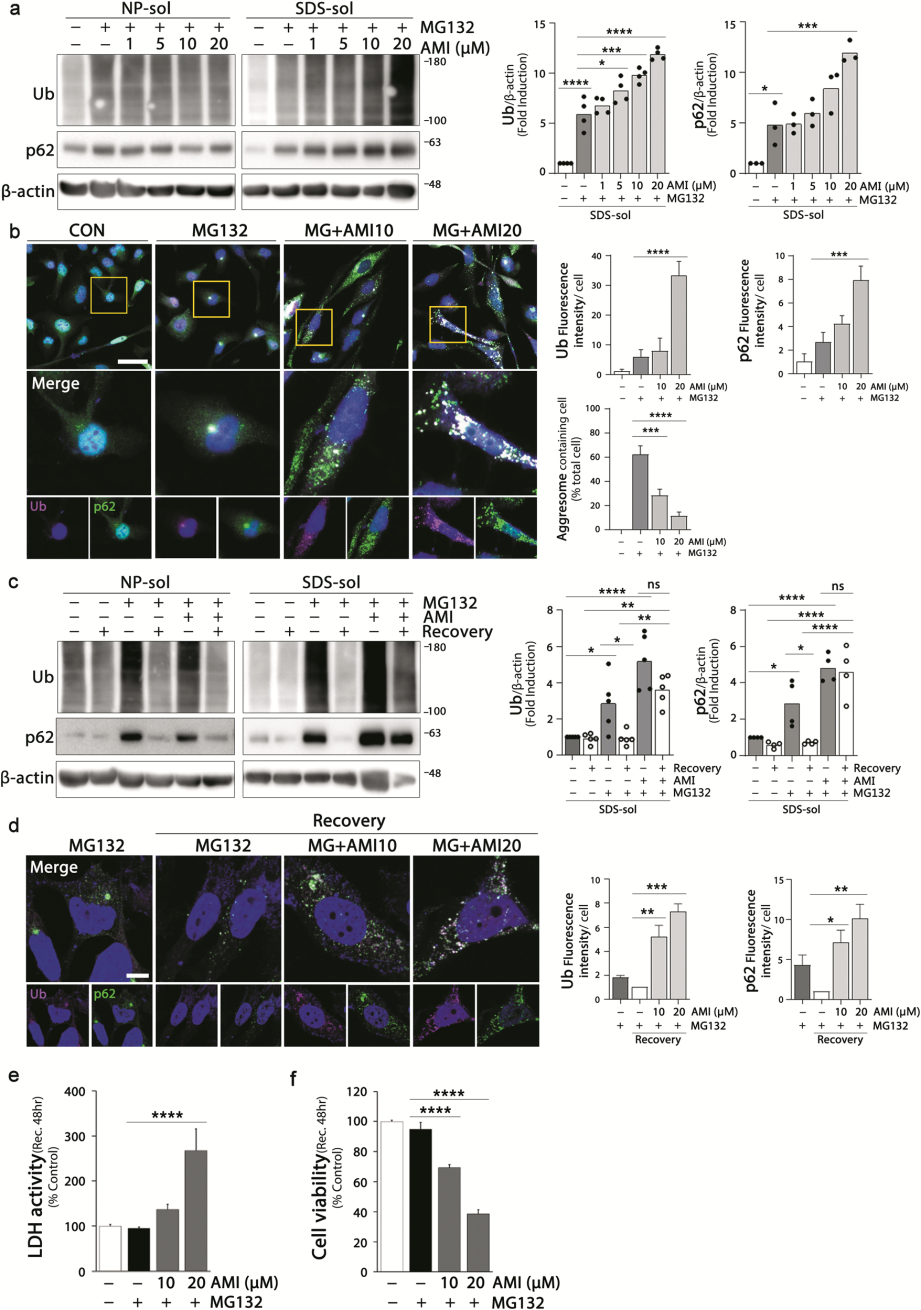
Effect of amitriptyline on MG132-induced accumulation of aggregates. **a**, **b** Ubiquitin and p62 levels in differentiated SH-SY5Y cells treated with amitriptyline and MG132 were analyzed by western blotting. SH-SY5Y cells were treated with MG132 in a two-step process (pro-incubated with a low dose of 0.1 μM for 16 h and changed to fresh normal media; after 8 h, incubated with a high dose of 1 μM for additional 16 h). Amitriptyline was applied 1 h prior to MG132 high-dose treatment. Quantification of ubiquitin and p62 in the SDS-fractions in **a** is shown in the right panel (*n* = 3–4). The expression and localization of ubiquitin (magenta) and p62 (green) in SH-SY5Y cells treated with MG132 and amitriptyline were analyzed by immunofluorescence (**b**). Scale bar corresponds to 50 μm. Quantification of ubiquitin and p62 immunofluorescence intensity in **b** is shown in the right panel. Percentage of aggresome (spots larger than 2 μm in diameter) in total cell is shown in the right panel (*n* = 60–100) **c** SH-SY5Y cells were treated with 1 μM MG132 in a two-step process and 20 μM amitriptyline as described in (**a**) and incubated with fresh media for an additional 24 h after MG132 treatment. Amitriptyline groups were treated amitriptyline in fresh media. Ubiquitin and p62 protein levels were analyzed by western blot. Protein levels in the SDS-fraction were quantified by densitometric analysis (*n* = 4–5). **d** The ubiquitin and p62 aggregates in MG132 and amitriptyline co-treated cells after the recovery period was evaluated by immunofluorescence. Scale bar corresponds to 10 μm. (*n* = 25–30) Quantification of ubiquitin and p62 immunofluorescence intensity in (**d**) is shown in the right panel. **e**, **f** SH-SY5Y cells were pretreated with amitriptyline for 1 h and treated MG132 in a two-step process, followed by a recovery period for 48 h. Cell death was assessed by LDH assay (**e**) and MTT assay (**f**) (mean ± S.E.M.; *n* = 7–8). *P < 0.05, **P < 0.01, ***P < 0.001, ****P < 0.0001. AMI amitriptyline, CON control, NP-sol NP-soluble fraction, SDS-sol SDS-soluble fraction, Ub ubiquitin.

Next we would like to verify whether amitriptyline exacerbates the accumulation of already existing protein aggregates in neuronal cells. We used a previously established in vitro experimental model that represents the accumulation of aggresome-like aggregates and following autophagy-mediated clearance of aggregates^34^. Significant increases in protein aggregates were detected in differentiated SH-SY5Y cells by sequential treatment with proteasome inhibitor MG132 (treated with 0.1 μM MG132 for 16 h, incubated with fresh media for additional 8 h, and then treated with 1 μM MG132 for 16 h). MG132-induced increases in ubiquitin and p62 levels in SDS-soluble fractions were exacerbated by amitriptyline (Fig. 3a). Amitriptyline itself induced accumulation of protein aggregates in neuronal cells (Fig. 1a and Supplementary Fig. S2a), and significantly aggravated the accumulation in MG132-treated cells.

Immunofluorescence images also showed that amitriptyline increased the MG132-induced accumulation of ubiquitin and p62 and their colocalization with increasing concentration, which was significant at 20 μM (Fig. 3b). In addition, amitriptyline significantly attenuated aggresome formation in MG132-treated cells. Clear and large immunospots (spots larger than 2 μm in diameter) of ubiquitin and p62, indicative of the formation of aggresome-like aggregates^34^, were detected in the cytosol of MG132-treated cells. However, in cells co-treated with amitriptyline and MG132, smaller and more scattered aggregates were distributed in the cytosol, instead of clear perinuclear aggresomes.

Because aggresome formation is necessary for the autophagy-mediated protein clearance, these findings suggest the possibility that amitriptyline interferes with autophagy-mediated protein clearance. To verify this, we examined the effect of amitriptyline on the clearance of MG132-induced protein aggregate in SH-SY5Y cells. After sequential treatment with the proteasome inhibitor MG132 to induce autophagy, the differentiated SH-SY5Y cells were subjected to recovery through fresh media for 24 h, and the protein aggregates levels were evaluated. During the recovery period, amitriptyline was added at the same concentration without MG132. As shown in Fig. 3c, d, almost all the ubiquitin and p62 proteins were removed in MG132-treated control cells after the recovery period, but not in cells treated with amitriptyline and MG132. Immunofluorescence showed that MG132-induced aggresome-like aggregates disappeared after the recovery period. In cells co-treated with amitriptyline and MG132, ubiquitin and p62 in the form of scattered spots throughout the cells were still detected after the recovery period. These results indicate that amitriptyline significantly interrupted the protein clearance system in MG132-treated cells even at 10 μM, and consequently, ubiquitinated proteins bound with p62 were not removed, but accumulated in the cytoplasm.

To evaluate whether disrupted protein clearance makes cells more vulnerable to MG132 toxicity, we examined the effect of amitriptyline on the viability of MG132-treated SH-SY5Y cells after a recovery period of 48 h (Fig. 3e, f). The viability of MG132-treated cells slightly decreased compared with that of control cells, whereas that of cells treated with 20 μM of amitriptyline and MG132 decreased to 38.55% compared with that of control cells (38.55 ± 2.80%, *P* < 0.0001 vs. untreated control cells; Fig. 3f). These results suggest that amitriptyline aggravates the accumulation of abnormal protein aggregates in the SH-SY5Y cells by disturbing the protein clearance system.

### Amitriptyline interferes with autophagy turnover

**Fig. 4:**
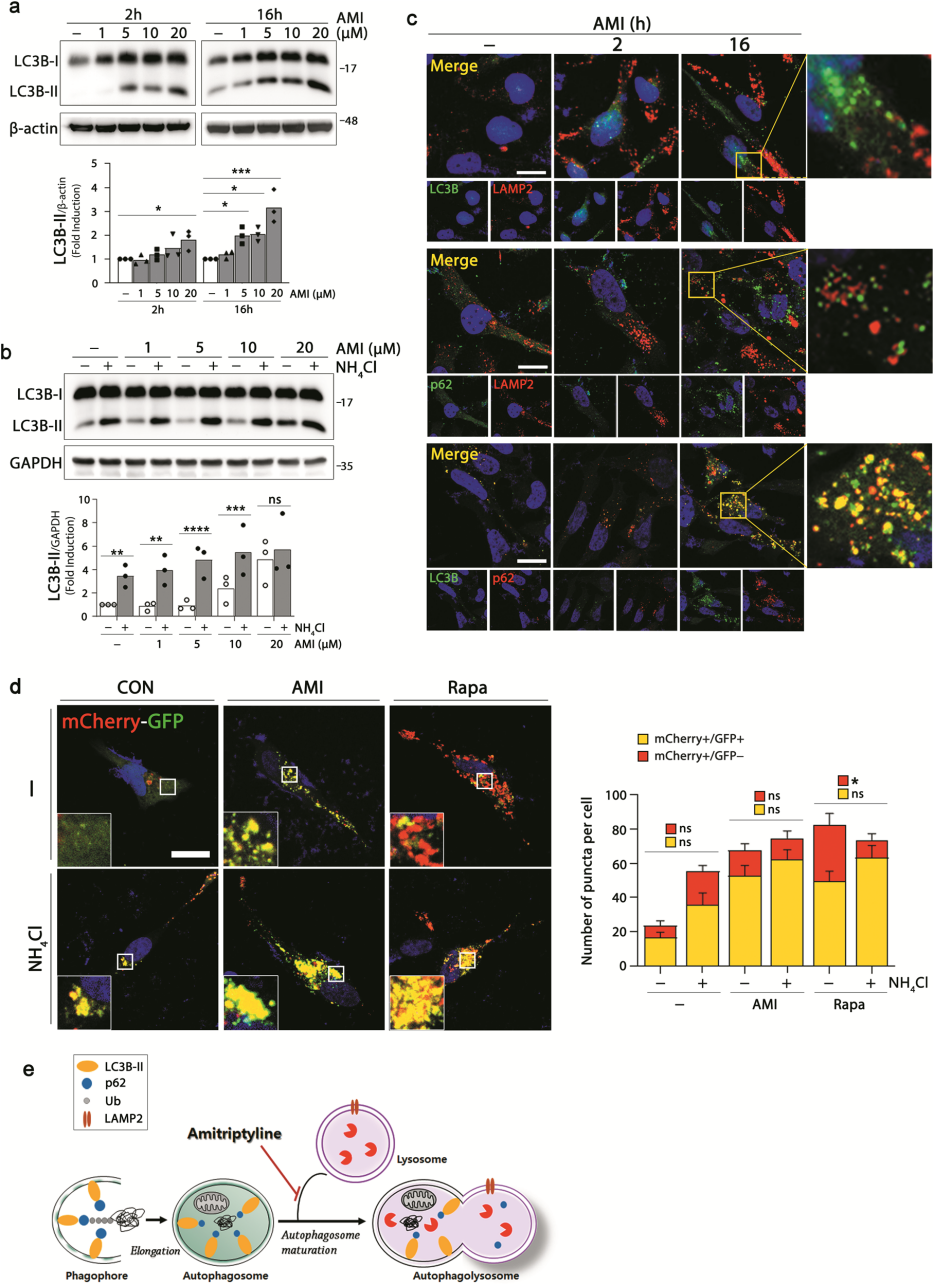
Inhibition of autophagy turnover by amitriptyline. **a** Differentiated SH-SY5Y cells were treated with amitriptyline (1, 5, 10, and 20 μM) for 2 and 16 h, and analyzed LC3B-I and LC3B-II levels by western blotting. LC3B-II levels were quantified by densitometric analysis (*n* = 3). **b** Cells were pretreated with 5 mM NH_4_Cl for 1 h, followed by amitriptyline (1, 5, 10, and 20 μM) for 24 h, and LC3B-I and II levels were analyzed by western blotting. Quantification of LC3B-II in (**b**) is shown in the below panel (*n* = 3). **c** Cells were treated with 20 μM amitriptyline for 2 or 16 h, and performed immunofluorescence against LC3B (green) and LAMP2 (red) in the first line, p62 (green) and LAMP2 (red) in the second line, and LC3B (green) and p62 (red) in the third line. Scale bar corresponds to 20 μm. **d** Cells were transfected with mCherry-GFP-LC3 plasmid and differentiated for 3 days. Cells were pretreated with 5 mM NH_4_Cl for 1 h, and treated with amitriptyline (20 μM) or rapamycin (2.5 μM) for additional 16 h. Scale bar corresponds to 50 μm. Quantification of mRFP+/GFP+ and mRFP+/GFP− puncta per cell is shown in right panel (*n* = 8–12). **e** Schematic diagram predicted effect of amitriptyline on autophagolysosome formation. *P < 0.05, **P < 0.01, ***P < 0.001, ****P < 0.0001. AMI amitriptyline. AMI amitriptyline, CON control, Rapa rapamycin, Ub ubiquitin.

We next asked which stage of the autophagy process was affected by amitriptyline and therefore relevant to abnormal protein clearance in amitriptyline-treated cells. To identify the step in the autophagy process affected by amitriptyline, we first examined the effect of amitriptyline on the LC3-II shift, a marker for autophagosomal formation (Fig. 4a). The increase in LC3B-II was detected with increasing concentration of amitriptyline as early as 2 h after treatment. A significant increase in LC3B-II was induced at 20 μM after 2 h of amitriptyline treatment, but more significant increases were detected even at low concentrations (5 and 10 μM) after 16 h. Because these increases can also be caused by the blockade of the autophagy process, we evaluated autophagic flux by measuring LC3B-II turnover. We compared the LC3B-II levels in amitriptyline-treated SH-SY5Y cells in the presence or absence of NH_4_Cl. NH_4_Cl inhibits the fusion of autophagosome and lysosome, as well as the degradation of a group of autophagosomal cargoes, by preventing the acidification in the lysosome^35^. Therefore, it is used to monitor autophagic flux. As shown in Fig. 4b, the presence of NH_4_Cl significantly increased LC3B-II levels, without significant differences between control and amitriptyline-treated cells. Higher concentration of amitriptyline (20 μM) itself caused LC3B-II accumulation, and there were no significant differences in the LC3B-II levels of cells treated with 20 μM of amitriptyline in terms of the absence and the presence of NH_4_Cl. These results suggest that the increase in LC3B-II by amitriptyline could be due to the blockade of autophagic flux.

From these results, we examined whether amitriptyline interfered with the fusion of autophagosome and lysosome to form autophagolysosome. Cells were treated with amitriptyline, and the distribution patterns of autophagosome marker LC3B, autophagosomal substrate marker p62, and lysosome marker LAMP2 were evaluated by immunofluorescence analysis (Fig. 4c). Both LC3B and LAMP2 levels were increased by amitriptyline in a time-dependent manner, but most were not colocalized. Similar result was detected in immunofluorescence image against p62 and LAMP2. But large portion of LC3B and p62 immunoreactivities were colocalized in amitriptyline-treated cells. To confirm the effect of amitriptyline on the autophagolysosome formation, we conducted additional experiment using a tandem tagged mCherry-GFP-LC3B plasmid. mCherry-GFP-LC3B is widely used to monitor autophagic flux; mCherry and green fluorescent protein (GFP) retain each fluorescence in autophagosome, but GFP fluorescence is quenched in the low pH of autophagolysosome^36^. SH-SY5Y cells transfected with mCherry-GFP-LC3B plasmids were treated with amitriptyline in the presence or absence with NH_4_Cl. As shown in Fig. 4d, both mCherry and GFP showed up in amitriptyline-treated cells, and NH_4_Cl had no significant effect on the number of autophagosome (yellow puncta) among the total puncta in amitriptyline-treated cells, which indicates defective formation of autophagolysosome by amitriptyline. On the other hand, rapamycin, a positive regulator of the autophagy induction, increased the quenching of GFP (suggestive of the maturation of autophagosome), which was significantly interrupted by NH_4_Cl (Fig. 4d). These results demonstrate that amitriptyline does not inhibit autophagosome formation, but inhibits autophagic flux by interfering with the fusion of autophagosome and lysosome (Fig. 4e).

### Amitriptyline regulates lysosome positioning

**Fig. 5:**
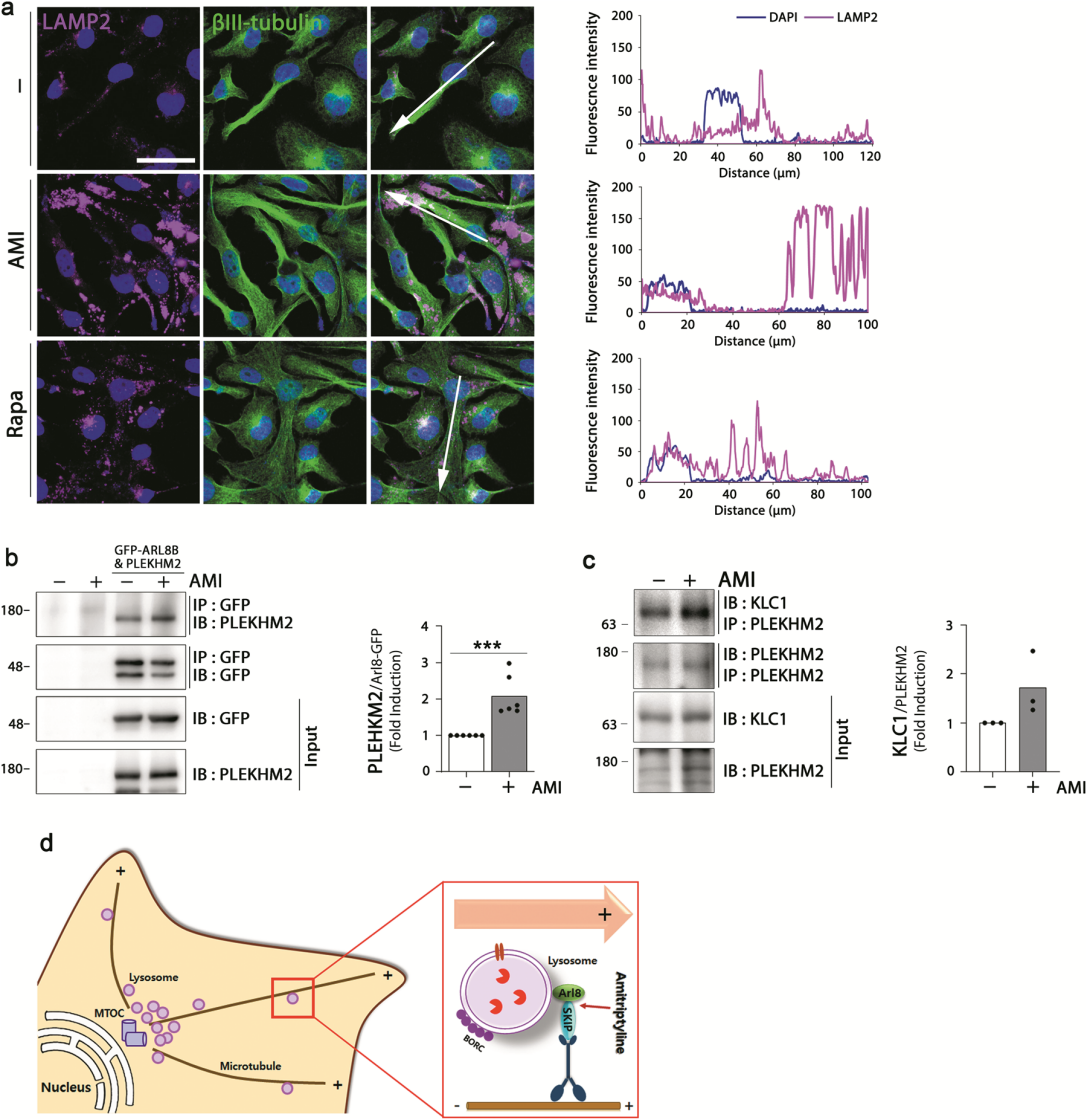
Effect of amitriptyline on lysosome distribution. **a** Differentiated SH-SY5Y cells were treated with amitriptyline (20 μM) or rapamycin (2.5 μM) for 24 h and analyzed LAMP2 and βIII-tubulin localization by immunofluorescence. Scale bar corresponds to 50 μm. Quantification of LAMP2 and DAPI intensity along the arrow line in **a** is shown in the right panel. **b** HEK-293T cells were transiently transfected with Arl8-GFP and PLEKHM2 and treated with 20 μM amitriptyline for 24 h. The binding of Arl8 and SKIP/PLEKHM2 in cells was analyzed by immunoprecipitation with anti-GFP and immunoblotting with anti-PLEKHM2. Quantification of PLEKHM2 per Arl8-GFP is shown in the right panel (*n* = 6). **c** HEK-293T cells were treated with amitriptyline for 24 h and the binding of KLC1 and PLEKHM2 in cells was analyzed by immunoprecipitation. Quantification of KLC1 per PLEKHM2 is shown in the right panel (*n* = 3). **d** Schematic diagram showing the effect of amitriptyline on lysosome positioning. ***P < 0.001. AMI amitriptyline, IP immunoprecipitation, IB Immunoblotting, Rapa rapamycin.

Lysosomes are highly dynamic organelles and their spatiotemporal characteristics could affect autophagolysosome formation and cargo degradation^37^. It is of interesting that immunofluorescence images of amitriptyline-treated SH-SY5Y cells showed that LAMP2 was mostly located at the distal end of cells compared with that of the control (Fig. 4c). To verify this, we compared the cytosolic localization of LAMP2 between cells treated with amitriptyline and positive autophagy regulator rapamycin (Fig. 5a). Large portion of LAMP2 immunoreactivity was detected in the peripheral part of amitriptyline-treated cells, but most of LAMP2 was detected in the perinuclear location of rapamycin-treated cells. Because SKIP (SifA and kinesin-interacting protein, also called PLEKHM2) and small GTPase Arl8 (Arf-like G protein) interact with kinesin and are relevant to the anterograde movement of the lysosomes^38^, we then determined whether the increased binding of Arl8, kinesin-1, and SKIP was involved in the peripheral distribution of lysosomes in amitriptyline-treated cells. We overexpressed HEK-293T cells with GFP-Arl8 and SKIP and performed immunoprecipitation for Arl8 and SKIP in cell lysates treated with 20 μM of amitriptyline. As shown in Fig. 5b, the interaction between SKIP and Arl8 was significantly increased by amitriptyline. Interaction between kinesin LC-1 (KLC1) and SKIP was also showed tendency to increase after treatment with amitriptyline (Fig. 5c). These results suggest that amitriptyline induces the translocation of lysosome to the plus-end direction of the cells by increasing the interactions between Arl8, kinesin, and SKIP (Fig. 5d).

### Amitriptyline activates PI3K/Akt/mTOR pathway

**Fig. 6:**
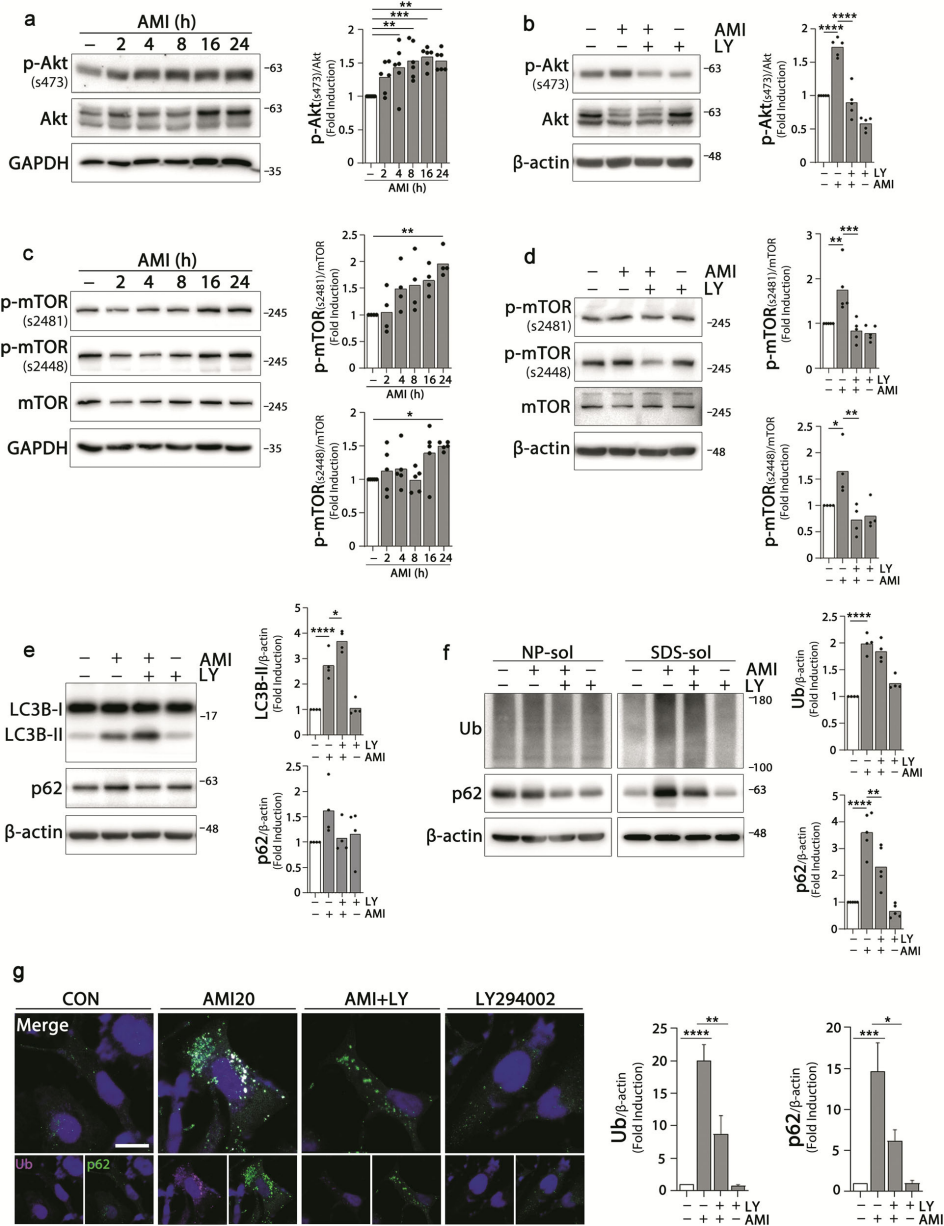
Activation of PI3K/Akt/mTOR pathway by amitriptyline. **a**, **c** Differentiated SH-SY5Y cells were treated with 20 μM amitriptyline for 2, 4, 8, 16, and 24 h, and the expression levels of p-Akt(S473), Akt, p-mTOR(S2481), p-mTOR(S2448), and mTOR were analyzed by western blotting. Quantification of each protein in (**a**, **c**) is shown in the right panel (*n* = 4–6). **b**, **d** Cell were pretreated with 10 μM PI3K inhibitor LY294002 for 1 h and treated with 20 μM amitriptyline for additional 24 h, and analyzed the protein levels as in (**a**, **c**) by western blotting. Quantification of each protein in **b**, **d** is shown in the right panel (*n* = 5). **e** LC3B and p62 levels in SH-SY5Y cells treated with amitriptyline in the presence or absence with LY294002 as **b**, **d** were analyzed by western blotting, and the quantification of protein is shown in the right panel (*n* = 4). **f** SH-SY5Y cells were treated LY294002 and amitriptyline, and the ubiquitin and p62 protein levels in NP- and SDS-soluble fractions were analyzed by western blotting. Protein levels in the SDS-fraction were quantified by densitometric analysis (*n* = 4–5). **g** Cells were treated with LY294002 and amitriptyline, and analyzed ubiquitin and p62 levels and localization by immunofluorescence. Scale bar corresponds to 20 μm. Quantification of ubiquitin and p62 immunoreactivities in (**g**) is shown in the right panel (5 independent sample, *n* = 15–25 cells) *P < 0.05, **P < 0.01, ***P < 0.001, ****P < 0.0001. AMI amitriptyline, CON control, LY LY294002, NP-sol NP-soluble fraction, SDS-sol SDS-soluble fraction, Ub ubiquitin.

PI3K/Akt/mTOR signaling pathway is a well-known upstream signaling pathway of autophagy^39^. Activated Akt phosphorylates and activates mTOR, which leads to the inhibition of autophagy through the phosphorylation of multiple autophagy-related proteins and blocks the elimination of aggregates. In this study, as shown in Fig. 6a–d, the levels of phospho-Akt(s473) were increased in cells treated with amitriptyline, which resulted in the increase of downstream signaling molecules such as p-mTOR(s2481) and p-mTOR(s2448**)**. Pretreatment with PI3K inhibitor LY294002 blocked the activation of Akt-mTOR signals induced by amitriptyline. Activated mTOR phosphorylates the UNC-51 like kinase (ULK) at serine 758 in human cell and consequently inhibits autophagy^40^. Amitriptyline increases p-ULK(s758), which was inhibited by LY294002 treatment (Supplementary Fig. 3a).

To verify the role of the activation of PI3K/Akt/mTOR in the blockade of autophagic flux and in the accumulation of protein aggregates in amitriptyline-treated SH-SY5Y cells, we treated cells with LY294002 and amitriptyline, and measured the levels of LC3B-II, p62, and ubiquitin. As shown in Fig. 6e, f, greater increases in LC3B-II levels and the attenuated accumulation of ubiquitin and p62 were detected in LY294002 co-treated cells compared with amitriptyline-treated cells. Immunofluorescence image also showed that amitriptyline-induced increase in ubiquitin and p62 immunoreactivities was significantly attenuated by LY294002 (Fig. 6g). These results suggest that the activation of the PI3K/Akt/mTOR pathway is responsible for the impaired autophagic flux observed in amitriptyline-treated cells.

### Amitriptyline induces Beclin 1 acetylation and interaction to Rubicon

**Fig. 7:**
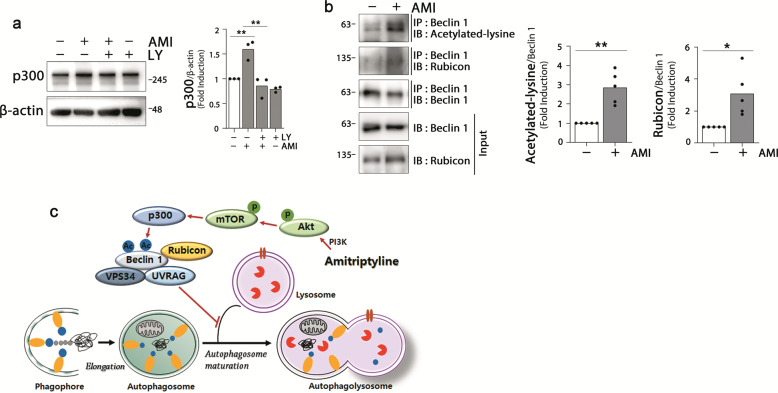
Effect of amitriptyline on Beclin 1 acetylation and Rubicon binding. **a** Differentiated SH-SY5Y cells were pretreated with 10 μM LY294002 for 1 h, followed by 20 μM amitriptyline for 24 h. p300 protein levels were analyzed by western blotting, and quantified by densitometric analysis (*n* = 3). **b** Cells were treated with 20 μM amitriptyline for 24 h, and analyzed protein interaction by immunoprecipitation with anti-Beclin 1 and immunoblotting with anti-Beclin 1, anti-acetylated-lysine, and anti-Rubicon, and quantified by densitometric analysis (*n* = 5). **c** Schematic diagram representing amitriptyline-mediated inhibition of autophagosome maturation and related signaling pathway. *P < 0.05, **P < 0.01. AMI amitriptyline, LY LY294002, IP immunoprecipitation, IB immunoblotting.

It has been suggested that Akt plays a crucial role in p300 regulation; Akt-induced phosphorylation of p300 is critical for the transactivation of p300^41^, and Akt/protein kinase B(PKB) is a positive regulator of the p300 expression^42^. To verify the involvement of Akt-linked p300 regulation in the amitriptyline-induced autophagy dysfunction, we would like to determine whether amitriptyline upregulates or activates p300, and Akt is responsible for the regulation. p300 was upregulated in amitriptyline-treated cells but not in cells co-treated with LY294002, indicating that amitriptyline activates Akt and subsequently, positively regulates p300 (Fig. 7a).

Recent studies have shown that Beclin 1 can be acetylated by p300 acetyltransferase, and acetylated Beclin 1 promotes the recruitment of Rubicon and inhibits autophagosome maturation and endocytic trafficking^43,44^. To verify this, we examined the effect of amitriptyline on the acetylation of Beclin 1. The amount of the acetylated-lysine protein binding to Beclin 1 was measured by immunoprecipitation of SH-SY5Y cell lysates treated with amitriptyline for 24 h. As shown in Fig. 7b, the acetylated Beclin 1 level was significantly increased in amitriptyline-treated cells. Simultaneously, a significant increase in Rubicon binding with Beclin 1 was also detected. These results suggest that amitriptyline induces Beclin 1 acetylation and Rubicon binding, and interferes with the fusion of autophagosome and lysosome (Fig. 7c).

## Discussion

In the present study, we showed that amitriptyline increases the accumulation of abnormal protein aggregates in neuronal cells and in the brain. Amitriptyline blocks autophagic flux by regulating the intracellular distribution of lysosomes, thereby resulting in the inhibition of autophagosome maturation.

Autophagy is a catabolic pathway that degrades unfolded proteins and damaged organelles. Throughout our lives, our bodies are exposed to a variety of stress factors both externally and internally, which result in protein modifications and accumulation of misfolded proteins. As the protein clearance system that controls protein quality becomes dysfunctional with aging, more and more protein aggregates are accumulated^45,46^. Interestingly, recent studies have demonstrated that basal autophagy is implicated in the regulation of neurogenesis in mature brain^47^, which might play a potential role in neurodegenerative pathologies. Therefore, autophagy dysfunction is closely related to the pathogenesis of neurodegenerative diseases^48–51^, and drugs targeting autophagy induction have a potential to prevent or ameliorate neurodegenerative diseases^52,53^. Conversely, it is also speculated that drugs or environments that inhibit autophagy might be risk factors for the development of neurodegenerative diseases^54^.

Amitriptyline is a TCA drug prescribed for regulating depressive symptoms. Conflicting results have been reported regarding its effects on autophagy function and on the development of neurodegenerative diseases. Several studies have shown that amitriptyline increases LC3B-II and Beclin 1 in neuronal cells^55^, augments the degradation of long-lived proteins through class-III PI3 kinase-dependent pathway^56^, and prevents neuronal degeneration in rotenone-induced PD mice model^57^. On the other hand, some studies have demonstrated that amitriptyline induces dopaminergic cell damage and causes PD-associated neurotoxicity^9^, and blocks the proliferation and angiogenesis in vascular endothelial cells by inhibiting autophagic flux^58^.

In this study, we verified the effect of amitriptyline on autophagy-mediated protein clearance and its relevant signaling pathways in neuronal cells. Clinically, amitriptyline is usually prescribed 75–150 mg/day, which corresponds to 0.18–0.5 µM levels in plasma after 6 weeks of treatment^59^. In animal study using mice, most pharmacological dose of amitriptyline to relief depressive symptoms is ranged 10–30 mg/kg (i.p.), and intraperitoneal LD_50_ (mouse) is 65 mg/kg (SAFETY DATA SHEET, Cayman). In this study, we injected amitriptyline (i.p. daily for 30 days; 5, 10, and 20 mg/kg) to male C57BL/6 mice, and showed tendency to increase in protein accumulation in the brain as low as 10 mg/kg, which was more significant in mice treated with 20 mg/kg. In vitro neuronal cell culture system, amitriptyline does not cause cytotoxicity up to 20 μM (Fig. 1) and IC_50_ for cell proliferation is reported to be 35.59 ± 3.48 μM^60^. To evaluate whether amitriptyline accumulation through long-term and/or high-dose use could be associated with the increased risk of neurodegenerative disease, we used relatively high concentrations in this study.

Autophagy is a complex and dynamic process; therefore, it is often difficult to distinguish between induction and inhibition. Autophagy begins with the nucleation of an isolated membrane that elongates to envelop target proteins into a double-membrane vacuole autophagosome and matures by the formation of autolysosomes^61^. The increased LC3-II level is most commonly used as an autophagy induction marker, but it also suggests impaired autophagic flux^62^. LC3-II levels are increased by amitriptyline, which may be due to the inhibition of autophagy turnover in SH-SY5Y cells. These are evidenced by our significant observations: (1) there was no significant difference in LC3B-II levels in amitriptyline-treated cells in terms of the presence or absence of NH_4_Cl (Fig. 4b); (2) autophagosome marker LC3B and p62 were colocalized, but not LC3B/or p62 and lysosome marker LAMP2, suggestive of disrupted autophagolysosome formation (Fig. 4c); and (3) no quenching of GFP was observed in cells transfected with mCherry-GFP-LC3B plasmid, suggesting defective formation of autophagolysosome (Fig. 4d). All these findings suggest that amitriptyline inhibits autophagy turnover rather than autophagy initiation.

The immunoreactivity of LAMP2 was increased by amitriptyline but was not colocalized with LC3B (Fig. 4). Interestingly, almost all LAMP2 were detected at the distal end of the cells (Fig. 5). The location of lysosome could be affected by environmental circumstances, such as pH and nutrition, and kinesin or dynein direct the lysosome through the microtubule to the plus or minus direction^63^. The Arf-like Arl8 is a small G protein that is a major regulator of components related to endocytic and secretory pathways^64^. Arl8 recruits kinesin-1 to lysosomes by binding to SKIP and migrates lysosomes to the cell periphery^38,65,66^, which suggests that Arl8 and SKIP constitute a core link between lysosomal membranes and kinesin-1. In this study, we demonstrated for the first time that the increased interaction between Arl8, SKIP, and kinesin could be responsible for the transport of the lysosomes at the distal end of amitriptyline-treated cells.

The PI3K/Akt/mTOR is one of the typical pathways modulating autophagy function. Akt is phosphorylated by PI3K, followed by the phosphorylation of mTOR, thus resulting in autophagy inhibition^39^. Several studies have shown that amitriptyline increases the phosphorylation of Akt^67,68^. Consistent with this, we found that amitriptyline activated Akt and mTOR, and the protein accumulation due to amitriptyline was attenuated by pre-treatment with PI3K inhibitor LY294002. mTOR activation plays a crucial role in the regulation of autophagy induction^69,70^, but evidences for the involvement of mTOR in the regulation of autophagic flux are conflicting^71,72^. Studies have shown that mTOR directly phosphorylates acetyltransferase p300 to regulate autophagy^73^. The fusion of autophagosome and lysosome to form autophagolysosome is critical for autophagy-mediated protein degradation. Beclin 1, a mammalian ortholog of yeast Atg6, interacts with several cofactors (e.g., Atg14L, UVRAG, Bif-1, Rubicon, Ambra1, HMGB1, nPIST, VMP1, SLAM, IP3R, PINK, and survivin) and regulates autophagy^74^. When Beclin 1 forms the Beclin 1–VPS34–UVRAG complex, autophagy is activated by autophagosome maturation. On the other hand, Beclin 1 can be acetylated by p300 acetyltransferase, and the Beclin 1–VSP34–UVRAG complex then binds to Rubicon, eventually interfering with the fusion of autophagosome and lysosome^44,75,76^. In this study, acetylated Beclin 1 was increased in amitriptyline-treated cells, and its interaction with Rubicon was also increased (Fig. 7).

In summary, our study shows that amitriptyline causes an accumulation of protein aggregates in neuronal cells through autophagy dysregulation. Amitriptyline does not block the initiation of autophagy, but blocks the autophagy turnover. Amitriptyline inhibits autophagic flux through (1) the activation of PI3K/Akt/mTOR pathway, (2) the activation of p300 and Beclin-1 acetylation, and (3) the increased interaction of Arl8b, SKIP, and kinesin, which moves the lysosome to the periphery of the cells and thus interferes with autophagosome fusion. The accelerated accumulation of abnormal aggregates could directly increase the risk of neuronal cell death in both healthy persons and those with already abnormal accumulation of protein aggregates. Therefore, our findings suggest that long-term and/or high-dose administration of amitriptyline might be associated with the increased risk of neurodegenerative disease.

## Materials and methods

### Antibodies and reagents

Dulbecco’s modified Eagle’s medium (DMEM), fetal bovine serum (FBS), penicillin/streptomycin, and trypsin/EDTA were purchased from Corning (Corning, NY, USA). Alexa Fluor^®^-conjugated secondary antibody, 4'6-Diamidino-2-Phenylindole, Dilactate (DAPI), and ProLong^™^ Gold antifade reagent were purchased from Invitrogen (Carlsbad, CA, USA). MG132 was from Enzo Life Sciences (Farmingdale, NY, USA). The protease and phosphatase inhibitor cocktails were obtained from Roche (Mannheim, Germany). The following antibodies were used: anti-LAMP-2 (H4B4, sc-18822), anti-p62 (D-3, sc-28359), anti-ubiquitin (P4D1, sc-8017), anti-ARL8A/B (H-8, sc-398635), anti-BECN1 (E-8, sc-48341), anti-phospho-p70 S6 kinase α (A-6, sc-8416), anti-p300 (F-4, sc-48343), anti-GFP (B-2, sc-9996), anti-TH (F-11, sc-25269), all obtained from Santa Cruz Biotechnology, Inc. (Santa cruz, CA, USA); anti-mTOR (2972S), anti-phospho-mTOR (Ser2448, 2971S), anti-phospho-mTOR (Ser2481, 2974S), anti-Akt1 (C73H10, 2938S), phospho-Akt (Ser473, 9271L), anti-beclin-1 (3738), anti-acetylated-lysine (9441S) from Cell Signaling Technology (Danvers, MA, USA); and anti-LC3 were purchased from MBL International (PM046, Woburn, MA, USA) and Novus Biologicals (NB600–1384, Littleton, CO, USA). Anti-GAPDH (G9545) and anti-β-actin (A1978) were from Sigma-Aldrich (St. Louis, MO, USA) and anti-α synuclein was from GeneTex (Irvine, CA, USA). Anti-phospho-ULK1(Ser758, PA5-78251), goat anti-Rabbit IgG (H + L) secondary antibody HRP (31460), and goat anti-Mouse IgG (H + L) secondary antibody HRP (31430) were purchased from Thermo Fisher Scientific (Waltham, MA, USA). All other chemicals including amitriptyline were of reagent grade and were purchased from Sigma-Aldrich.

### Cell culture

Human neuroblastoma SH-SY5Y cells and HEK-293T cells were grown in DMEM containing 10% FBS, 100 IU/L penicillin, and 10 μg/mL streptomycin at 37 °C in a humidified atmosphere with 5% CO2 and 95% air. The cells were plated on polystyrene culture dishes at a density of0.055 × 105 cells/well in a 48-well culture plate, 0.19 × 105 cells/well in a 6-well culture plate, or 0.1 × 106 cells in a 60-mm dish. SH-SY5Y cells were differentiated by incubation for 3 days with 10 μM all-trans retinoic acid and 1% FBS in DMEM. LUHMES cells were cultured in precoated dishes with 50 μg/mL poly-l-ornithine hydrobromide and 1 μg/mL fibronectin (Sigma-Aldrich). Cells were maintained in DMEM/F12 containing 1× N2 supplement and 40 ng/mL recombinant basic fibroblast growth factor (bFGF) (Thermo Fisher). For differentiation, cells were changed to the differentiation medium consisting with DMEM/F12, 1× N2 supplement, 1 mM dibutyryl cAMP, 1 μg/mL tetracyclin (Sigma-Aldrich), and 2 ng/mL recombinant human GDNF (R&D systems, Minneapolis, MN, USA). After 5 days of differentiation, cells were treated with amitriptyline in fresh proliferation medium. For primary cultures of cortical neurons, E18 C57BL/6 mice were dissected, as described previously^77^. Cortex tissues were incubated in StemPro^®^ Accutase^®^ Cell Dissociation Reagent (Gibco, Grand Island, NY, USA) for 10 min at 37 °C and washed twice with Hank’s Balanced Salt Solution. Dissociated tissues were triturated and resuspended in neurobasal medium (Gibco) with 1% of l-glutamine (100×; Gibco), 2% of B27 supplement (50×; Gibco), and 1% of penicillin–streptomycin solution (100×; Corning, NY, USA). Cells were plated at an appropriate density on 60 mm dish precoated with poly-d-lysine (Sigma-Aldrich). After incubation for 4 h, cells were changed with fresh media and maintained in a humidified CO_2_ incubator. At day in vitro 11, amitriptyline were treated for 24 h.

### Transient transfection

SH-SY5Y cells were transiently transfected with pDEST-CMV mcherry-GFP-LC3B (Addgene, Watertown, MA, USA**)** using polyethylenimine (PEI) reagents (Polyscience, Warrington, PA, USA) according to the manufacturer’s instructions. Transient transfection of HEK-293T cells with pDEST47-ARL8B-GFP (Addgene) and pCMV3-PLEKHM2 plasmids (Sino biological, Beijing, China) was performed using PEI reagents. pDEST47-ARL8B-GFP was deposited by the Richard Kahn Lab (Emory University Department of Biochemistry, Atlanta).

### Assessment of cell viability: lactate dehydrogenase assay

The extent of cell death was measured from the amount of lactate dehydrogenase released into the culture medium. Fifty microlitre of cell culture medium were incubated at room temperature (RT) with 100 mM potassium phosphate buffered saline (PBS) containing 23 mM pyruvate (pH 7.4) and 0.26 mM NADH with a total volume of 200 μL. The rate of NAD + formation was measured at 340 nm for 5 min at 25-s intervals using a microplate spectrophotometer (VersaMax; Molecular Devices, San Jose, CA, USA).

### Assessment of cell viability: 3-(4,5-dimethylthiazol-2-yl)-2,5-diphenyltetrazolium bromide (MTT) assay

Cells were plated in a 48-well tissue culture plate and incubated with yellow MTT solution at a final concentration 0.5 mg/mL for 4 h. Then, purple formazan crystals were dissolved in DMSO. The absorbance of the solubilized formazan product was measured at 570 nm using a microplate spectrophotometer (VersaMax; Molecular Devices).

### Cell fractionations and western blot analysis

Cells were lysed in NP buffer (150 mM NaCl, 1% NP-40, 1 mM EDTA, 5% glycerol, 25 mM Tris–Cl; pH 7.5) with protease and phosphatase inhibitor cocktails (Roche, Basel, Switzerland) for 60 min at −80 °C. Cell lysates were centrifuged at 15,000 rpm for 60 min at 4 °C, and the supernatant proteins in the NP-soluble fraction were collected. The pellet was further lysed in SDS buffer (2% SDS, 50 mM Tris–Cl; pH 7.5) with protease and phosphatase inhibitor cocktails (Roche, Basel, Switzerland), sonicated for homogenization, and boiled at 100 °C for 5 min. The SDS-soluble fraction was centrifugated at 15,000 rpm for 60 min at 4 °C, and the supernatant were collected. Protein samples were subjected to sodium dodecyl sulfate polyacrylamide gel electrophoresis (SDS-PAGE) and immunoblotted as previously described^34^.

### Western blot analysis

Cells were washed with ice-cold PBS, harvested, and lysed in radioimmunoprecipitation assay (RIPA) buffer (150 mM NaCl, 1% NP-40, 0.5% deoxycholic acid, 0.1% SDS, 50 mM Tris–Cl; pH 7.5) with complete protease and phosphatase inhibitor cocktails for 30 min at –20 °C. Cell lysates were centrifuged at 15,000 rpm for 30 min at 4 °C. Equal amounts of protein were separated by 8–12% SDS polyacrylamide gels and transferred onto polyvinylidene difluoride-nitrocellulose membranes (Millipore). The membranes were blocked with 5% skim milk in TBST and were incubated at 4 °C overnight with specific primary antibodies and then with HRP-conjugated horseradish peroxide-conjugated secondary antibody. Specific bands were detected by enhanced chemiluminescence (ECL; Millipore, Billerica, MA, USA) and analyzed by a luminescent image analyzer LAS-4000 (Fujifilm, Tokyo, Japan). The relative intensities of each band were measured using Image J software.

### Immunoprecipitation

Cell lysates were incubated with A/G agarose beads (Santa Cruz, sc-2003) for 1 h to eliminate non-specific binding proteins. After the preclearing step, the lysates were centrifugated at 2500 rpm for 5 min, and the supernatant were incubated with the indicated antibody for 1 h and with A/G agarose beads overnight. The beads were washed with IP buffer (0.025 M Tris, 0.15 M NaCl, 0.001 M EDTA, 1% NP-40, 5% glycerol; pH 7.4) 5 times and boiled with 2× loading dye for 10 min. The sample is loaded on SDS-PAGE for further analysis.

### Immunofluorescence confocal microscopy

SH-SY5Y cells were plated on poly-d-lysine-coated cover slips in 12-well culture plates. After the individual experiments, the cells were washed twice with PBS and fixed for 15 min in cold 4% paraformaldehyde and 4% sucrose in PBS (pH 7.4). After fixation, the cells were permeabilized with 0.25% triton-X-100 in PBS for 10 min and then blocked with PBS containing 1% bovine serum albumin (BSA), 1% normal donkey serum, and 3% FBS for 30 min at room RT to reduce non-specific binding. They were then incubated overnight with primary antibodies in PBS containing 1% BSA at 4 °C and with Alexa Fluor^®^-conjugated secondary antibodies for 50 min at RT, and DAPI was added for 10 min.

### Animal treatment

All experiments involving animals were approved by the Institutional Animal Care and Use Committee at CHA University (IACUC 170116). Male C57BL/6 mice, 6 weeks of age, were housed under a 12:12 light–dark lighting schedule with free access to food and water. The RT was 22 °C. The mice were injected intraperitoneally with sterile PBS (100 μL each, *n* = 5) or amitriptyline (5, 10, or 20 mg/kg in a volume of 100 μL PBS each, *n* = 5) daily for 30 days. After the last injection, the mice were euthanized with Zoletil 50 (Virbac) and transcardially perfused with PBS, heparin, and 4% PFA in PBS (pH 7.4).

### Rotarod test

Motor function of the mice was assessed using a rotarod system (Rota Rod-R V2.0, B.S. Technolab). Before the experiment, mice were trained on the rod (diameter, 3.5 cm) with an increasing speed 4 rpm for 3 min. After the training, the speed of rod increased from 4 to 40 rpm and retention time of each mouse on the rod was recorded, ≤300 s. Each animal was tested twice times and the final time records were used for statistical analysis.

### Immunohistochemistry

After perfusion, fixation was performed in 4% paraformaldehyde for more than 24 h and cryo-protected in 30% sucrose/0.1 M PBS solution for another 24 h followed by the freezing of tissues in optimal cutting temperature compound. Afterwards, 20 μm PFA-fixed cryo-sections were serially cut in the coronal plane for unbiased stereology, and brain sections were incubated for 30 min in 1% BSA, 0.2% Triton X, 0.3% H_2_O_2_, and 2% normal goat serum (NGS) in 0.05 M PBS. The sections were then incubated with mouse anti-p62/SQSTM1 or mouse anti-ubiquitin, diluted in 2% NGS in 0.05 M PBS overnight at 4 °C. After two rinses in 0.05 M PBS, the sections were incubated with secondary biotinylated goat anti-mouse (Vector Laboratories, Burlingame, CA) for 1 h and with ABC solution (Vector Laboratories) in 0.05 M PBS for 1 h and developed with 0.025% 3,3′-diaminobenzidine tetrahydrochloride (DAB; Sigma, St. Louis, MO).

### Statistical analysis

Data were analyzed and expressed as mean ± standard error of the mean. Statistical significance was determined by one-way ANOVA, followed by Bonferroni post hoc tests using Prism software (GraphPad Software; La Jolla, CA, USA). *P* < 0.05 was considered significant for all statistical analysis.

## Supplementary information

Supplementary Figure Legends

Supplementary Figure 1

Supplementary Figure 2

Supplementary Figure 3
