# Supplementary material for: Amitriptyline interferes with autophagy-mediated clearance of protein aggregates via inhibiting autophagosome maturation in neuronal cells
Source: Cell Death Dis. 2020 Oct 17;11(10):874. doi: 10.1038/s41419-020-03085-6 (PMC7568721; doi:10.1038/s41419-020-03085-6)
Supplement: Supplementary file 1 — Supplementary Figure Legends [file 41419_2020_3085_MOESM1_ESM.docx]

**Supplementary Materials: Amitriptyline interferes with autophagy-mediated clearance of protein aggregates via inhibiting autophagosome maturation in neuronal cells**

**Supplementary Figure Legends**

**Figure S1. Effect of amitriptyline on accumulation of alpha-synuclein in primary dopaminergic neuronal cells.** **a** Dopaminergic neuronal cells from E13.5 C57BL/6 mice were treated with amitriptyline at 20 μM for 48 h after day *in vitro* 10 (DIV10). The levels and distribution of α-synuclein (yellow) and TH (Red) were analyzed by immunofluorescence. Scale bar corresponds to 50 μm. AMI: amitriptyline, CON: control, α-SYN: α-synuclein, TH: tyrosine hydroxylase.

**Figure S2. Accumulation of aggregates in amitriptyline and MG132 treatment. a** Differentiated SH-SY5Y cells were 1 h pretreated with amitriptyline (1, 5, 10, and 20 μM) and MG132 in a two-step process. AMI: amitriptyline, NP-sol: NP-soluble fraction, SDS-sol: SDS-soluble fraction, Ub: ubiquitin.

**Figure S3. Effect of amitriptyline on downstream signals of PI3K/mTOR/AKT pathway.** **a** SH-SY5Y cells were pretreated with LY294002 at 10 μM for 1 h, followed by amitriptyline at 20 μM for 24 h. The expression levels of p-ULK(s758) and p-p70S6K were analyzed by western blotting. AMI: amitriptyline.
